# Composite Medical Tabletops Made of CFRP with Different Cross-Sections: Numerical Analysis and Laboratory Testing

**DOI:** 10.3390/ma16247574

**Published:** 2023-12-09

**Authors:** Przemysław Golewski, Daniel Pietras, Tomasz Sadowski, Albin Michał Wit-Rusiecki

**Affiliations:** 1 Department of Solid Mechanics, Faculty of Civil Engineering and Architecture, Lublin University of Technology, Nadbystrzycka 40 Str., 20-618 Lublin, Poland; p.golewski@pollub.pl (P.G.); d.pietras@pollub.pl (D.P.); 2 WIT-Composites Ltd., Bohdana Dobrzańskiego 3 Str., 20-262 Lublin, Poland; wit@wit-composites.com

**Keywords:** operating table, CFRP composite, numerical simulations

## Abstract

This paper presents the results of laboratory tests of CFRP (carbon fiber-reinforced polymer) laminates, which allowed the development of numerical material models. The obtained data were used in a further stage to perform numerical simulations of four variants of medical tabletops, differing, among other features, in the shape of the cross-section. Maximum deflections and effort in the composite material were analyzed. The final step was to perform a laboratory test for one of the tabletop versions, the results of which confirmed the correctness of the numerical calculations. This work is aimed at both researchers and designers involved in the practical application of fiber-reinforced polymer matrix composites.

## 1. Introduction

Basic equipment in the operating room includes a basin stand, trolleys, Mayo table, run-about buckets, diathermy apparatus, suction apparatus, anesthesia machine, drip stand, operating lights, viewing screens, swab rack, and an operating table [1]. There are many types of operating tables, but from the point of view of designing the tabletop itself, the following features are important:

- The tables are usually divided into three sections to support the body, allowing the patient to assume a bent or lying position,
- Extensions for the head are also used, e.g., for neurosurgical or endoscopic examinations,
- Many accessories are available, such as a hand support that attaches to movable clamps on the rail [2].

Another solution is the Jackson table method [3], which allows the patient to be transferred from the bed to the table, which is equipped with appropriate inserts. The frame is made of CFRP and sits above the patient. Once the patient is strapped to the table, the frame can be rotated to any angle depending on the examination being performed. This type of table provides flexibility in patient positioning, which is important, for example, in examinations for oblique lumbar intervertebral fusion [4].

Adjustable carbon-fiber operating tables used for lumbar spine surgery have a similar design. The flexion angle of the table should allow for increasing or decreasing the lumbar lordosis to access the disk space [5].

In addition to the design of the tabletop, a mechanism that allows it to tilt to a lateral position is also important. The authors in the article [6] presented the results of a study of the biomechanical effort of personnel performing a patient transfer from one table to another. With the use of a tilted tabletop, the effort of the pushing nurse was eliminated and the activity of most of the muscles of the pulling nurse decreased. Thus, the use of lateral tilt tops should also be included in numerical simulations, with attention paid to the mounting locations where the complex stress state occurs.

Tabletop joint failure situations can occur in practice; for example, when a patient is morbidly obese. Such an incident is reported in [7]. While transferring a patient weighing 130 kg from a surgical table to a bed, two bolts of the turning mechanism failed, almost causing the patient to fall. This type of situation is likely to increase as the incidence of obese patients in the operating room increases. This situation once again underscores that one of the important design points is the joint between the operating tabletop and the tilt mechanism column. Both pull-out and shear tests are required [8].

Another important design issue is the occurrence of pressure from different parts of the body. If a patient lies in one position for dozens of hours, pressure injuries called pressure sores develop. This topic was presented in [9], where pressure relieving pads were used at the interface between the patient’s body and the medical tabletop. Pressure at the junction of both heels and sacrum was measured in the supine position. Comfort was assessed using a visual analogue scale. The use of polyurethane foam helped reduce pressure and improve patient comfort. Thus, when designing the upper part of the medical top, some curvatures can be preliminarily introduced for greater patient comfort and against pressure sores.

A similar topic related to surface pressure is presented in [10]. Experimental studies were conducted on a total of 72 patients, divided into three groups differing in the material used to reduce pressure. The materials used were a gel support surface, a viscoelastic support surface, and a standard operating table. The pressures exerted on the patient’s body were lowest with the viscoelastic material, and this type of material is recommended to minimize the risk of pressure injury in the operating room.

In conclusion, composite medical tabletops are an important component of operating room equipment. Their design is related to both safety and comfort, as well as the prevention of overpressure injuries in contact with the patient’s body. However, there is a lack of articles in the literature on the process of designing and testing such composite products. The presented article will help fill this gap. 

The presented results are related to a scientific project with the title Composite system for fast mechanical connections for the medical industry. Using CAD modeling and numerical simulations, three types of original tabletops were proposed, which are relatively thin, ergonomically shaped, and free of filling. In the final analysis, a solution was chosen that represents a compromise: the tabletop is flat and has infill only near the edges and the attachment zone. The purpose of the work was to test the technology of making such a tabletop and compare the laboratory results with a numerical model. 

## 2. Materials and Methods

Polymeric materials have been used in medicine for more than 150 years. Their first appearance dates back to 1862 when Alexander Parker first presented a modified thermoplastic material at an exhibition in London [11]. The rapid dissemination of these type of materials in medicine was possible due to their advantages such as low cost, excellent mechanical properties, ease of sterilization, and bactericidal properties. In addition, they have weak magnetic properties and much less absorption of X-rays, which makes them irreplaceable in certain applications [12,13]. To increase their strength, the structure is reinforced, which leads to the formation of a composite. The literature [14] lists several types of composites that can be used in medical devices. These include composites reinforced with short fibers or flakes spaced irregularly or in a specific direction, glass beads, powders, or the use of continuous fibers resulting in laminates. Each of these reinforcing materials causes changes in the properties of the polymer material, e.g., resulting in reduced shrinkage, and increased strength and stiffness.

Medical equipment is divided into 3 classes [14,15] depending on the application and the safety and risks involved. The first class applies to such devices as tongue depressors, bandages, gloves, and bedpans. The second class includes, for example: wheelchairs, X-ray machines, MRI machines, surgical needles, catheters, and diagnostic equipment. The last group includes such products as heart valves, stents, implanted pacemakers, silicone implants, and hip and bone implants. Thus, operating tables and their tops will fall into the second class of medical devices, while the materials from which they are made must be tested for their strength.

### 2.1. Laboratory Tests

Laboratory tests were performed to determine the elastic and strength properties of composite materials to build their numerical model. The tests were made for two types of materials:

- Composite reinforced with unidirectional fibers—KORDCARBON-CPREG-200-UD-3K-EP-120-38 (UD),
- Composite reinforced with twill weave fabric—KORDCARBON-CPREG-245-T-3K-EP1.5-42-T (TW),
- Both types of materials use an epoxy matrix and carbon fibers and were produced by Fiberpreg GmbH, Carl Zeiss Str. 7, 89231 Neu-Ulm, Germany.

All test samples were made by Wit Composites using a vacuum bag autoclave technology [16] and epoxy matrix prepregs [17].

Tensile and compression tests were made for both types of materials. A summary of all tests, taking into account the angle of the fibers concerning the direction of loading (0°, 45°, 90°), is presented in Table 1.

**Figure A1:**
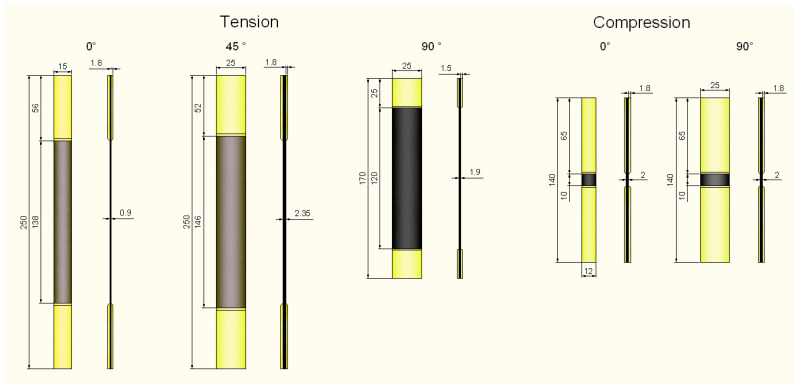
Shapes and dimensions of samples made of KORDCARBON-CPREG-200-UD-3K-EP-120-38 (UD).

**Figure A2:**
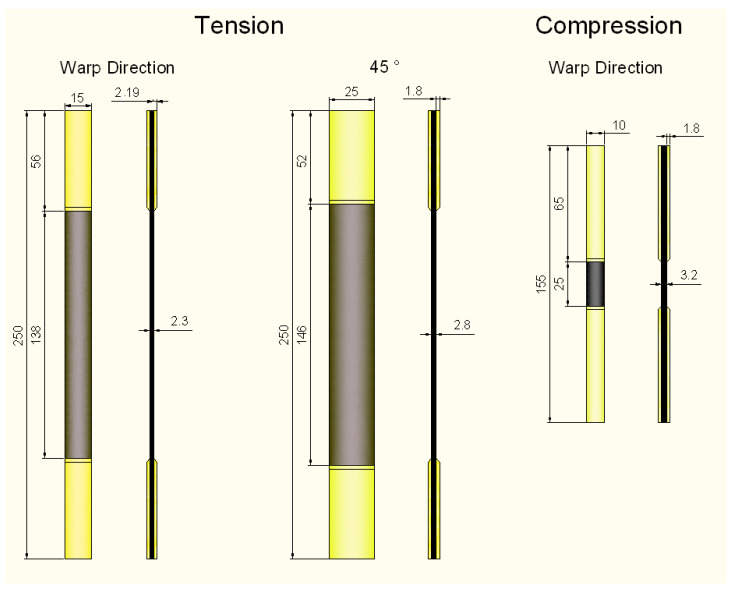
Shapes and dimensions of samples made of KORDCARBON-CPREG-245-T-3K-EP1.5-42-T (TW).

The tests were made by the following standards:

- D3518/D3518M—13 Standard Test Method for In-Plane Shear Response of Polymer Matrix Composite Materials by Tensile Test of a 45° Laminate [18],
- Designation: D3039/D3039M—14 Standard Test Method for Tensile Properties of Polymer Matrix Composite Materials [19],
- D6641/D 6641M—Standard Test Method for Determining the Compressive Properties of Polymer Matrix Composite Laminates Using a Combined Loading Compression (CLC) Test Fixture [20].
- The shapes of the used samples with dimensions are shown in Appendix A, Figure A1 and Figure A2.

**Figure A3:**
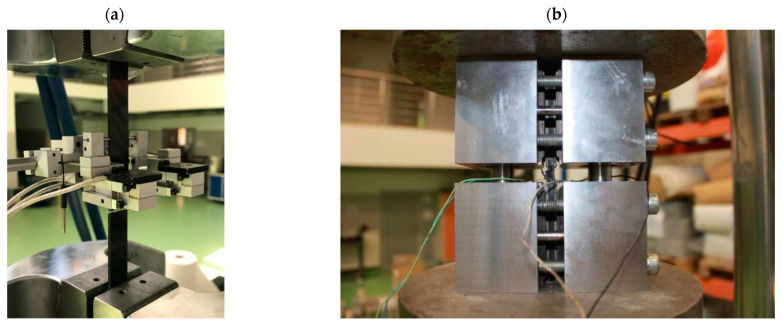
Test stand: (**a**) Tensile test, (**b**) Compression test.

Tensile tests were made using an MTS810 machine equipped with a 250 kN force measuring head. Loads were applied at a constant speed of 2 mm/min, whose strain rate was 0.6 min^–1^ (less than 2.5 min^–1^, which satisfies the requirement of quasi-static loading [21]). Strain measurements were made with a biaxial extensometer. The test stand is shown in Figure A3a.

For compression tests, MTS810 testing machine equipped with a special platform for clamping specimens was also used. Loading forces were measured using a load cell with a range of 250 kN. The test was made under conditions of constant displacement speed. The speed of 0.4 mm/min (strain rate 0.18 min^–1^), was selected to meet the test-to-failure time condition, which should be in the range of 1 to 10 min. In this case, electro-resistance strain gauges with a base of 3 mm were used for strain measurements. Strain gauge measurements were made using a HBM MGC PLUS type amplifier with CATMAN application. The test stand is shown in Figure A3b.

Normal stresses σ [MPa] were determined from the following equation:

(1) $$\sigma =\frac{F}{h\cdot w}$$

where: F—loading force [N], h—sample thickness [mm], w—sample width [mm]. The tangential stress τ_12_ [MPa] was determined from the following equation:

(2) $$\tau_{12}=\frac{F}{2\cdot h\cdot w}$$

The longitudinal modulus of elasticity E [GPa] was determined as follows:

(3) $$E=\frac{\sigma_{0}-\sigma_{1}}{\varepsilon_{a0}-\varepsilon_{a1}}$$

where ε_a0_ denotes the initial strain of the elastic modulus determination range of 1000 μm/m, while ε_a1_ denotes the final strain of the elastic modulus determination range of 3000 μm/m. The symbols σ_0_ and σ_1_ are the stress values corresponding to strains with the same subscript from the beginning and end of the range in which the longitudinal elastic modulus was determined.

The Poisson’s ratio was determined as the ratio of the difference between transverse and longitudinal strains in the same range used to determine the longitudinal modulus of elasticity.

(4) $$\nu =\frac{\varepsilon_{t0}-\varepsilon_{t1}}{\varepsilon_{a0}-\varepsilon_{a1}}$$

For ±45° samples, the shear strain of γ_12_ was determined as the difference of the measured longitudinal and transverse strains as follows:

(5) $$\gamma_{12}=\varepsilon_{a}-\varepsilon_{t}$$

The modulus of elasticity G_12_ was determined as the ratio of the increase in shear stress to the corresponding increase in shear strain (6) according to document D3518/D3518M—13, this modulus was determined in the range of shear strain 2000 μm/m–6000 μm/m.

(6) $$G_{12}=\frac{\tau_{12}^{0}-\tau_{12}^{1}}{\gamma_{12}^{0}-\gamma_{12}^{1}}$$

The shear strength of the tested materials was determined as the stress that occurred at a shear strain equal to 5%.

The standard deviation was calculated using Equation (7):

(7) $$\sqrt{\frac{\sum {\left(x-\bar{x}\right)}^{2}}{n-1}}$$

where: *x* is the value of a parameter for a given sample, $\bar{x}$ is the average value of a given parameter for all samples, and n is the number of samples tested.

The differences between the parameters were determined as follows:

- If an increase in the parameter is observed:

(8) $$\left(\frac{x_{k}}{x_{p}}-1\right)\cdot 100\%$$

- If a decrease in the parameter is observed:

(9) $$\left(1-\frac{x_{k}}{x_{p}}\right)\cdot 100\%$$

The tests were performed at an average temperature of 27.7 °C and a humidity range of 49.9–51.4%. Measurements of environmental parameters were made once a day around 8:30 a.m.

### 2.2. Numerical Simulations

Numerical simulations were made using Abaqus software 6.16. S4R-type shell parts were used for the CFRP laminate. S4R element is the reduced integrated quadrilateral finite-membrane-strain element which adopts reduced integration theory advocated to treat the shear lock. There were also solid parts in model 4, e.g., foam filling or aluminum mounting plate for which C3D8R-type elements were used. The C3D8R element is a general purpose linear brick element, with reduced integration (1 integration point). 

In the case of shell parts for laminates, it was possible to apply the Tsai–Hill composite strength criterion [22]. Before that, it was necessary to define the following values:

- Tensile strength in the fiber direction (X_t_),
- Compressive strength in the fiber direction (X_c_),
- Tensile strength in the direction perpendicular to the fibers (Y_t_),
- Compressive strength in the direction perpendicular to the fibers (Y_c_),
- Shear strength (S).

The Tsai–Hill hypothesis has the following form:

(10) $$\frac{\sigma_{11}^{2}}{X^{2}}-\frac{\sigma_{11}\sigma_{22}}{X^{2}}+\frac{\sigma_{22}^{2}}{Y^{2}}+\frac{\tau_{12}^{2}}{S^{2}}=1$$

where:

σ_11_—normal stress in the direction of the fibers,

σ_22_—normal stress in the direction perpendicular to the fibers,

τ_12_—tangential stress,

X—strength in the fibers’ direction,

Y—strength in the direction perpendicular to the fibers,

S—shear strength.

If a value of 1 is reached in the material, the element will thus be completely efforted. The criterion in the form (10) also remains valid when the composite material has different tensile and compressive strength characteristics. Then, the modification of this criterion is to insert in place of X and/or Y, depending on the sign of the stresses σ_11_, σ_22_—values of tensile strength X_t_, Y_t_ or compressive strength X_c_, Y_c_. 

**Figure 1:**
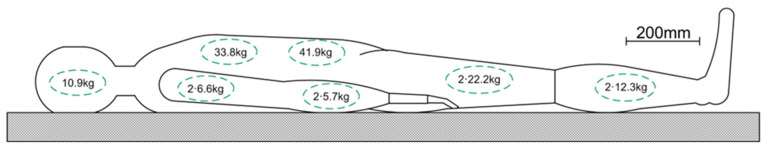
Distribution of weights from the patient’s body.

Figure 1 shows the values of loads that are taken for strength calculations of tabletops. Based on them, the corresponding partitions at the top of the tabletop were separated, which made it possible to set loads.

The calculations were performed for a total of four tabletops, differing in cross-section, layer arrangement, and external dimensions. The simulations were divided into two main groups:

- Ergonomic tabletops (without making a demonstrator),
- Tabletop with a rectangular cross-section (made demonstrator).

**Figure 2:**
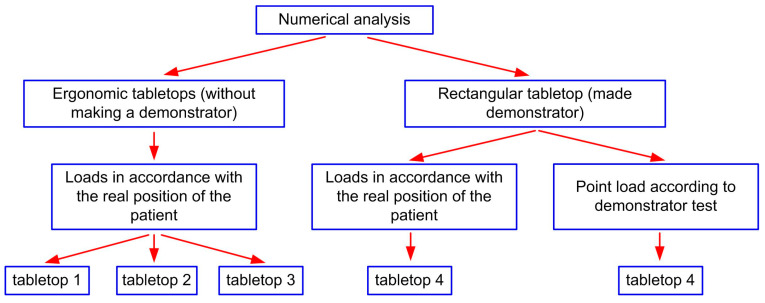
Classification of numerical simulations.

To better illustrate the scope of performed work, it is shown in the diagram in Figure 2.

**Figure 3:**
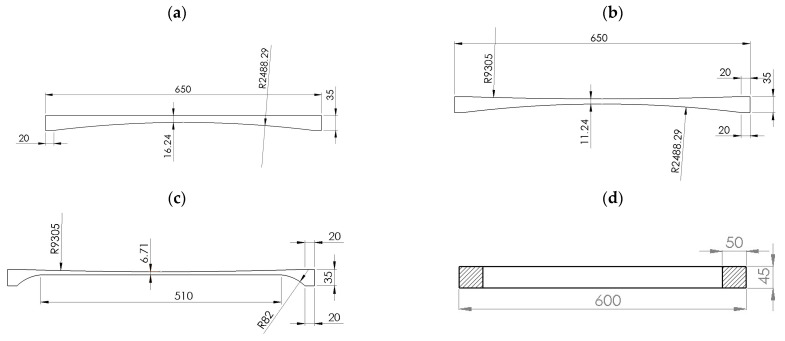
Cross-sections of the analyzed tabletops: (**a**) Tabletop 1, (**b**) Tabletop 2, (**c**) Tabletop 3, (**d**) Tabletop 4.

The external dimensions of the ergonomic tabletops (1–3) were 2100 mm × 650 mm, while the external dimensions of the rectangular tabletop 4 were 2300 mm × 600 mm. Because the tabletop should be as transparent to X-rays [23] as possible, models 1 through 3 were made as no-fill. Partial filling was used in tabletop 4. Usually, polyurethane foam is used for this purpose, which adversely affects the translucency of the tabletop during imaging with the “C” arm [24], hence the width of the filling at the edges of the tabletop is only 50 mm. Cross-sections of the analyzed tabletops are shown in Figure 3.

**Figure 4:**
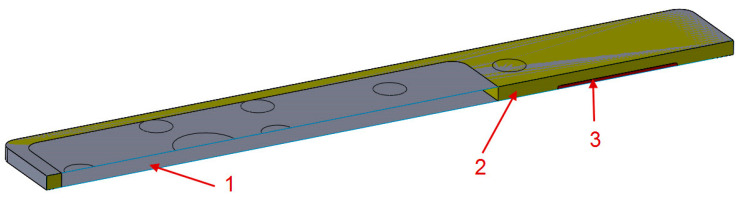
Longitudinal cross-section of tabletop 4. 1—CFRP laminate, 2—Polyurethane foam, 3—Aluminum plate.

Total filling was used in a section of tabletop 4 (Figure 4) because of the mounting to the column. Depending on the imaging site, the patient can be positioned either head over the column or legs over the column, whereas higher loads will be achieved in the second position.

The same cross-section of the wall was used for models 1 through 3. The wall consisted of 15 layers. Both twill fabric (TW) and unidirectional (UD) fibers were used. In both cases, these were carbon fibers. The arrangement of the layers was as follows: 3 × TW, 2 × UD, 1 × TW, 3 × UD, 1 × TW, 2 × UD, 3 × TW, with the UD fibers arranged along the longer edge of the tabletop. 

For model 4, a different arrangement of layers was used: 1 × TW, 4 × UD, 2 × TW, 4 × UD, 1 × TW, making a total of 12 layers. 

The purpose of simulations 1 through 3 was to determine what effect the introduction of an ergonomic shape has by using a radius to keep the patient’s body on the axis of the tabletop. Demonstrators were not made for this type of tabletop, since the introduction of radiuses requires the manufacture of appropriate molding tools, which significantly increases costs.

Tabletop number 4 has a rectangular cross-section and is therefore much easier to manufacture. A demonstrator was made for this case to verify the numerical calculations. 

**Figure 5:**
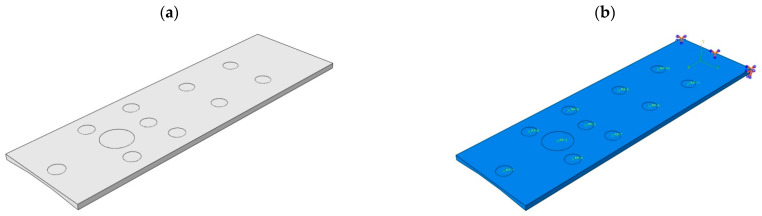
Models 1–3: (**a**) Performed partitions, (**b**) Boundary conditions.

Because the weight of the body is distributed unevenly over the entire surface, a partition was made as in Figure 5a. 

The top of the operating table can be treated as a cantilever plate. For models 1 through 3, one end (on the lower limb side) was fixed—all degrees of freedom were taken away, while the other end was free. Reference points were placed in the made partitions and connected to them. This was to make it easier to apply loads in the form of concentrated forces (Figure 5b). Each layer of laminate was arranged along the axis of the tabletop.

**Figure 6:**
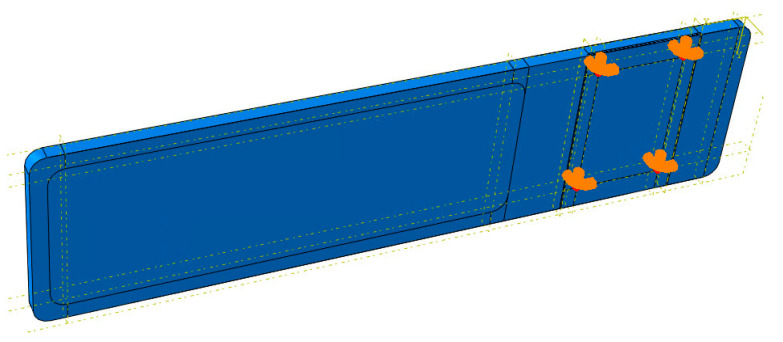
Method of mounting tabletop 4.

For model 4, as mentioned above, a demonstrator was made, so the method of attachment cannot be theoretical as in the case of models 1–3. For model 4, an aluminum plate was used at the bottom (Figure 4), the purpose of which was to transfer the load from the tabletop to the point fasteners to the column. The boundary conditions used for the model can be seen in Figure 6 (orange shapes). All degrees of freedom have been taken away in the four holes of the aluminum plate; this is analogous to the use of bolts at these locations.

**Figure 7:**
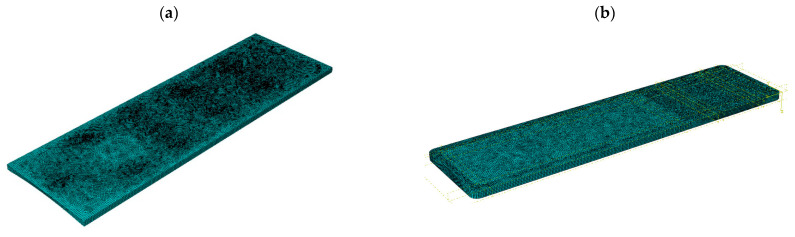
Finite element meshes: (**a**) Models 1–3, (**b**) Model 4.

After the necessary partitions were made, FEM meshes were applied as shown in Figure 7. For models 1 to 3, about 42,000 S4R elements were used, while for model 4 a total of 91,703 elements were used, including 36,635—S4R, 1022—S3 (CFRP laminate), and 54,046—C3D8R (polyurethane foam and aluminum plate).

## 3. Results of Material Testing

**Figure 8:**
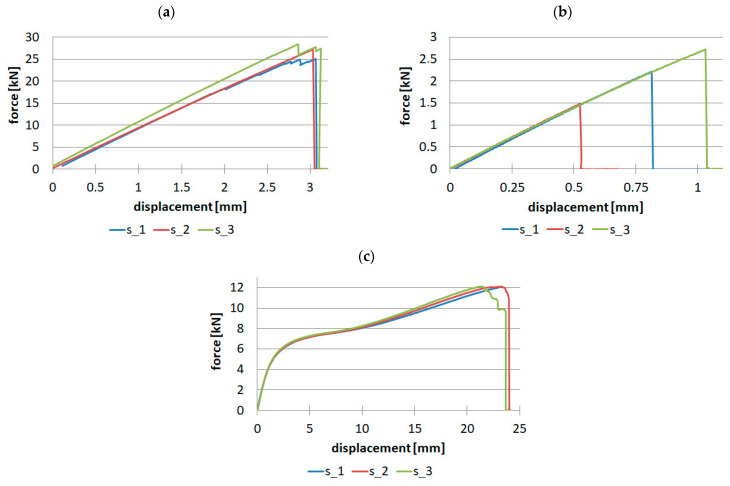
Tensile results for unidirectional reinforced samples: (**a**) 0° direction, (**b**) 90° direction, (**c**) 45° direction.

Material research for uniaxial tensile tests for samples made of unidirectional fiber-reinforced prepreg was performed on a batch of three samples. The tests were performed in three directions: 0, 90, and 45 degrees. Force-displacement diagrams are shown in Figure 8.

This type of test made it possible to determine the following elastic properties:

E_t0_ = 121.965 GPa—Young’s modulus in the direction of fiber orientation,

E_t90_ = 7.817 GPa—Young’s modulus transverse to the direction of fiber orientation,

G_12_ = 3.434 GPa—Kirchhoff’s modulus,

ν_12_ = 0.298—Poisson’s ratio,

And strength properties:

X_t_ = 2003.119 MPa—tensile strength in the direction of fiber orientation,

Y_t_ = 45.12 MPa—tensile strength transverse to fiber orientation,

S = 58.749 MPa—shear strength.

The relative standard deviation analysis is noteworthy. For samples with a fiber arrangement along the tensile direction, a value in the range of 6.37–6.5% was obtained for tensile strength and Young’s modulus, and a much lower value of 2.68% was obtained for Poisson’s ratio. For samples with a fiber orientation perpendicular to the tensile direction, low repeatability of results was obtained for maximum force, and thus for tensile strength. The relative standard deviation was as high as 29.2%, while for Young’s modulus, it was 1.67%. Considering the tests for samples with fibers oriented at an angle of 45 degrees, the values of relative standard deviation were in the range of 1.15–3.6% for shear strength and Kirchhoff modulus, respectively. 

**Figure 9:**
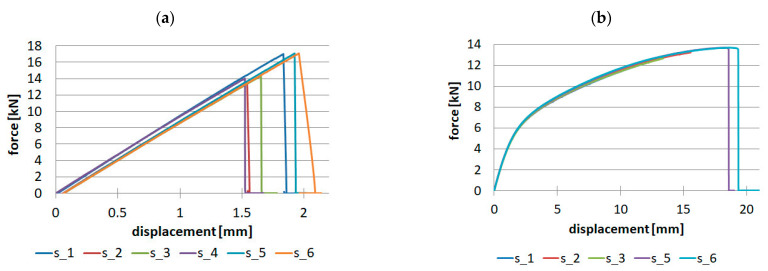
Tensile results for the fabric: (**a**) Warp direction, (**b**) 45° direction.

For samples made of twill fabric, two tests were necessary for the direction along the warp orientation and at an angle of 45 degrees. The results are presented in Figure 9.

This type of test made it possible to determine the following elastic properties:

E_t0_ = 48.069 GPa—Young’s modulus in the direction of warp orientation,

G_12_ = 2.652 GPa—Kirchhoff’s modulus,

ν_12_ = 0.064—Poisson’s ratio,

And strength properties:

X_t_ = 453.385 MPa—tensile strength in the direction of warp orientation,

S = 58.730 MPa—shear strength.

For fabric-reinforced samples, it was assumed that E_0_ = E_90_ and X_t_ = Y_t_. For performed tests, the maximum value of relative standard deviation was obtained for tensile strength—10.86%. The smallest values were obtained for tests with a 45-degree fiber orientation: for shear strength 1.64% and Kirchhoff modulus 1.58%. 

**Figure 10:**
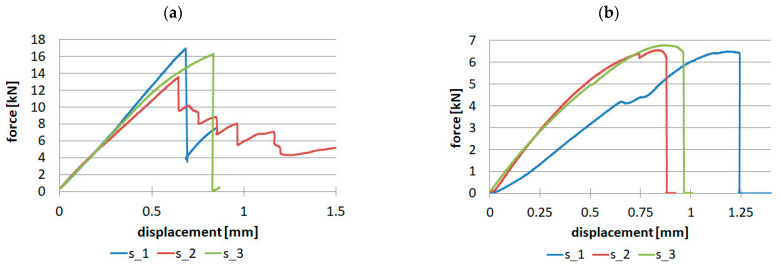
Results for unidirectional fibers for compression: (**a**) 0° direction, (**b**) 90° direction.

The composite tabletops work similarly to a cantilevered plate, so the upper part is in tension and the lower part is in compression. Hence, to make the numerical simulations more in line with reality, compression tests were also carried out, the results of which for the composite reinforced with unidirectional fibers are shown in Figure 10.

Performed tests allowed us to determine the following elastic properties:

Ec_0_ = 111.564 GPa—Young’s modulus in the direction of fiber orientation,

Ec_90_ = 8.094 GPa—Young’s modulus transverse to the direction of fiber orientation,

And strength properties:

X_c_ = 793.504 MPa—compressive strength in the direction of fiber orientation,

Y_c_ = 131.592 MPa—compressive strength transverse to the direction of fiber orientation.

Analysis of the relative standard deviation showed that the largest value (10.9%) is for compressive strength for the direction along the fiber orientation, while the smallest value (0.69%) is for Young’s modulus values also for the direction along the fiber orientation. For the perpendicular direction, the relative standard deviation is in the range of 2.5–3.78% for Young’s modulus and compressive strength, respectively. 

**Figure 11:**
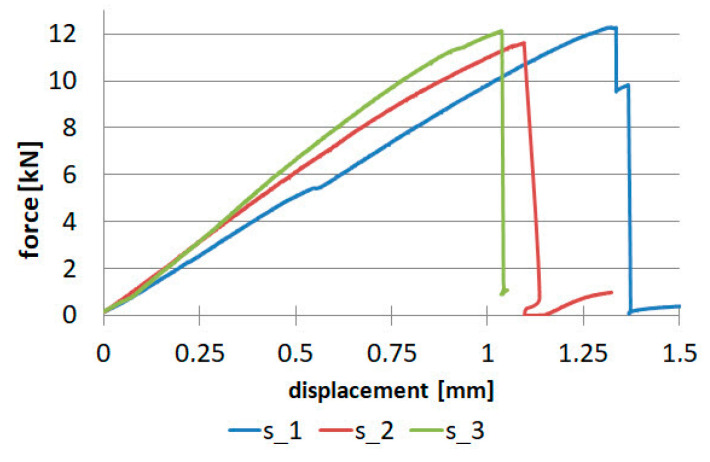
Results for fabric in compression.

The compression test was also carried out for the composite reinforced with twill fabric, only for warp direction, since we assume the same values for weft direction. The results for the batch of samples are shown in Figure 11.

As a result of the tests, Young’s modulus in compression Ec = 43.4 GPa and compressive strength Xc = 208.99 MPa were determined. The relative standard deviations for both of the above-mentioned parameters were 0.53% and 6.27%, respectively.

A summary of the results for all tests can be found in Appendix B.

## 4. Results of Numerical Simulations

**Figure 12:**
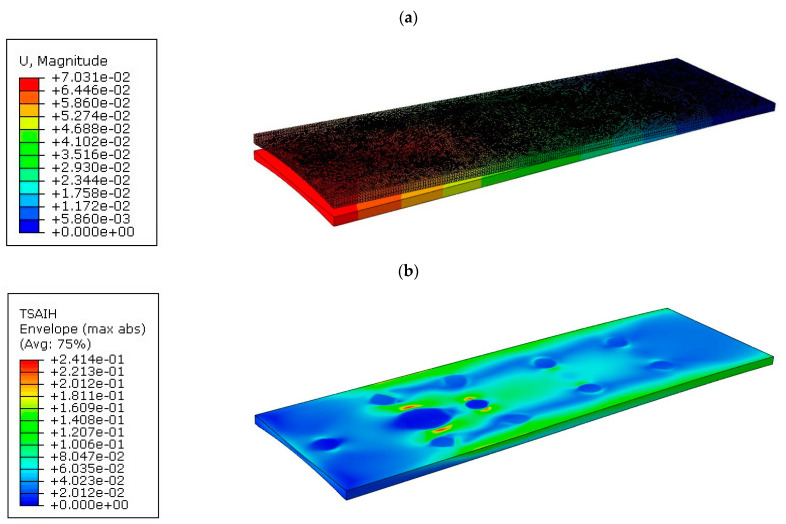
Results for tabletop 1: (**a**) Deflection, (**b**) Tsai–Hill criterion.

The results of the numerical simulations are presented in the form of resultant displacement maps “U, Magnitude” and the achievement of the Tsai–Hill strain criterion. The resultant displacement is calculated as the square root of the sum of the squares of the respective displacements along the *x*, *y*, and *z* axes. For the displacement maps, the state before deformation was also shown, and the deflection of the tabletops was not scaled. Figure 12a shows the displacement map for tabletop 1, with a maximum value of 70.31 mm.

Figure 12b shows the effort of the composite material for tabletop 1. The maximum value was 24.14%.

**Figure 13:**
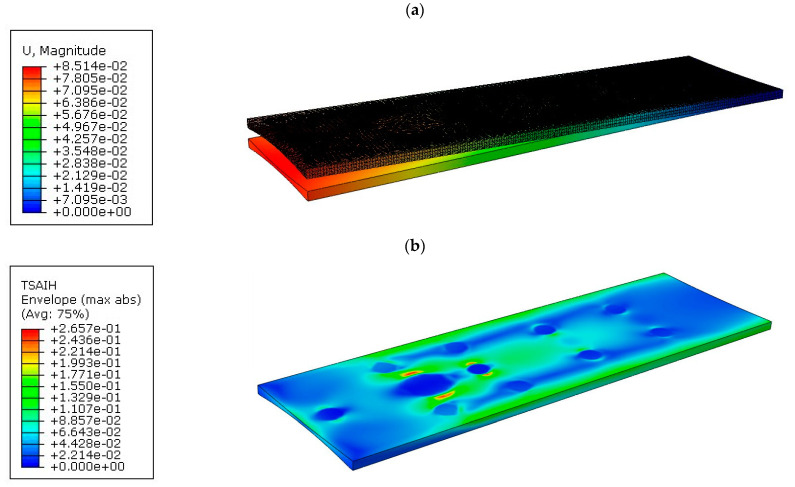
Results for tabletop 2: (**a**) Deflection, (**b**) Tsai–Hill criterion.

Tabletop 2 had two radiuses of curvature compared to the previous tabletop 1, hence the thickness thinning was even greater. This resulted in an increase in maximum deflection to a value of 85.14 mm (Figure 13a).

However, the introduction of two radiuses of curvature does not result in a significant increase in material effort. For tabletop 2, a maximum value of 26.57% was obtained, as shown in Figure 13b.

**Figure 14:**
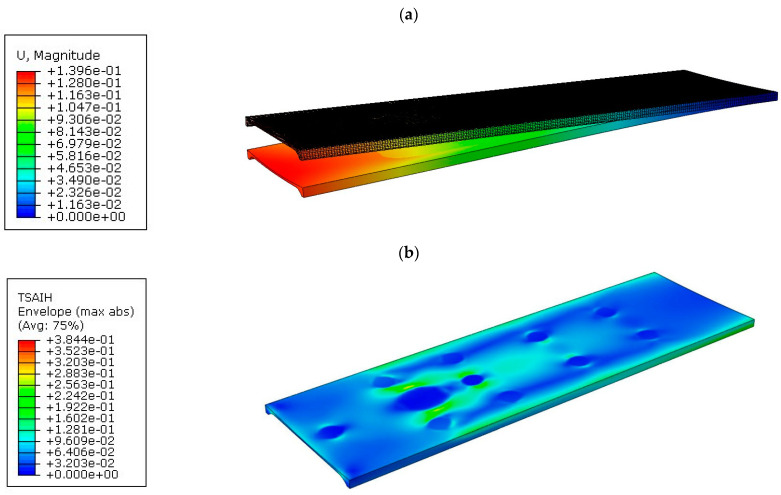
Results for tabletop 3: (**a**) Deflection, (**b**) Tsai–Hill criterion.

Tabletop 3 had one side flat, while the other side was shaped as a curve; the minimum thickness was only 6.71 mm and was almost constant across the width of the tabletop, hence the maximum displacement was as much as 139.6 mm (Figure 14a).

However, such a large displacement did not translate into a significant increase in material effort, which in this case was 38.44% (Figure 14b).

**Figure 15:**
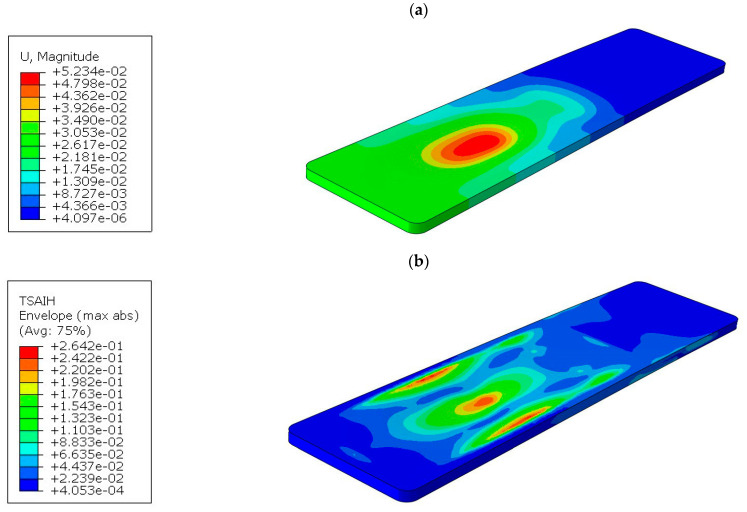
Results for tabletop 4: (**a**) Deflection, (**b**) Tsai–Hill criterion.

In all three cases, the effort of the material was not exceeded, but due to technological reasons, a rectangular cross-section was finally adopted for tabletop 4. The method of mounting is also different, as can be seen in the displacement map in Figure 15a.

The part of the tabletop on the side of the mounting to the column is unloaded, as indicated by the blue area. The maximum displacement of 52 mm occurs where the torso of the body is located. In real conditions, this value will be lower because the weight from the body is distributed more uniformly than shown in Figure 2. The tabletop, due to the maintenance of high translucency, has foam stiffeners only on the perimeter and in the area of attachment to the column, while the inside is hollow. The free end of the tabletop experiences a displacement of 26 mm. 

The material effort for tabletop 4 is shown in Figure 15b, with a maximum value of 26.4%, so the safety factor is almost 4. The maximum values are also concentrated in the torso area and in the zones near the edge where the laminate joins the foam filling.

## 5. Technological Test of Tabletop Bending

**Figure 16:**
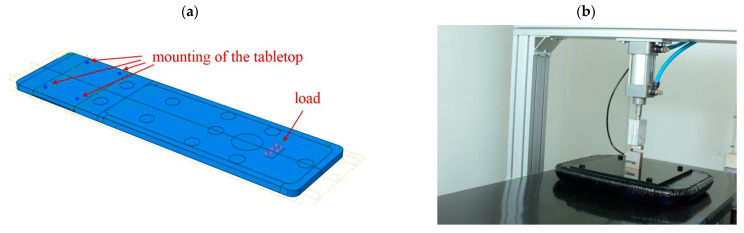
Testing of tabletop 4: (**a**) Mounting and loading method, (**b**) Laboratory test.

To be sure that the simulations were carried out correctly, both additional simulations and a laboratory test were performed. Because it is difficult to reproduce identical conditions as in Figure 1 with gradually increasing load at all points, it was decided to apply the load only at one point 30 cm away from the face of the tabletop for values from 5 to 50 kg with increments of 5 kg. The separated partition for the load in the numerical model can be seen in Figure 16a. Figure 16b shows a section of the tabletop with the sensor attached to a rigid aluminum frame. An intermediate element was placed between the sensor and the composite table in order to not induce stress concentrations [25]. 

In this way, the tabletop could be subjected to a deflection test precisely and without introducing vibrations or asymmetrical loads. A measuring cell was also attached to the pneumatic actuator so that the load could be precisely controlled. The load cell was attached to the actuator on one side, while the other side was pressed against a rigid plate, which in turn had a soft filling on the contact side of the tabletop so as not to cause significant pressure concentrations that could cause local damage to the laminate. The deflection of the tabletop was measured at its end using an electronic sensor. 

After the laboratory test, numerical calculations were made by modifying only the load location and its value in model 4.

Table 2 summarizes the results of deflection at the end of the tabletop with increments of 5 kg. The percentage differences between the laboratory test and numerical simulation are also shown.

**Figure 17:**
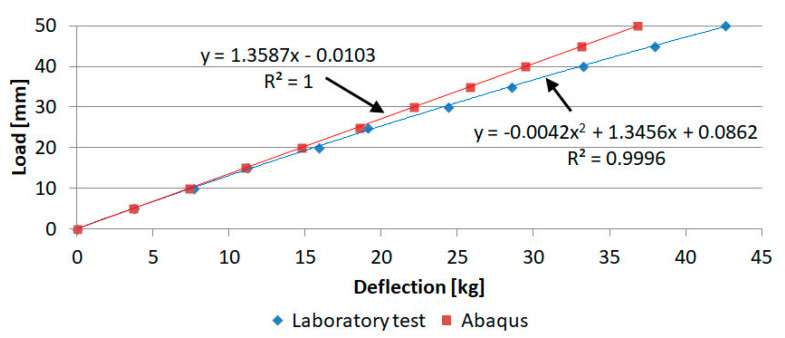
Comparison of results from laboratory test and numerical simulation for tabletop 4.

Percentage differences in results in the range of loads up to 25 kg range from 0.81–7.37%, while in the range from 25 kg to 50 kg they reach higher values in the range of 9.44–13.53% and tend to increase. This shows that the elastic response of the tabletop is not linear, which is also visible in Figure 17.

The laboratory test resulted in a value of 42.56 mm, while the numerical simulation resulted in a value of 36.8 mm. Therefore, the percentage difference concerning the laboratory test is:

$$\Delta =\frac{42.56-36.8}{42.56}\cdot 100\%=13.53\%$$

This is lower than the 20% that was used to determine the milestone in the project from which the research was funded. 

**Figure 18:**
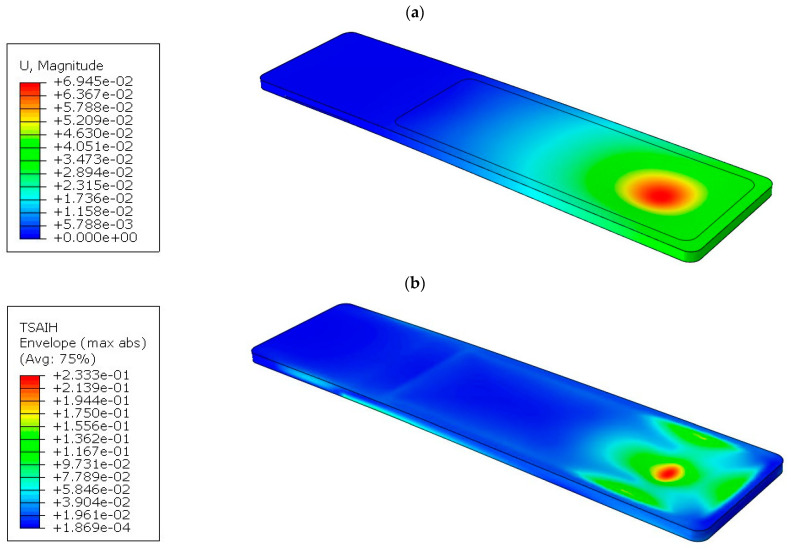
Results for tabletop 4 for point loading (50 kg): (**a**) Deflection, (**b**) Tsai-Hill criterion.

Figure 18a shows the displacement map for a maximum load of 50 kg.

In addition, the effort of the material was determined (Figure 18b). According to the Tsai–Hill hypothesis, it is at the level of 23.3%. Thus, even with a point load application of 50 kg, the safety factor is still high and exceeds the value of 4.

## 6. Discussion

An analysis of the literature has shown that there are currently no suitable procedures or examples for both designing medical tabletop structures and performing their simulation. As a guideline for design, it is possible to take tabletop loads considering the patient’s body and the corresponding safety factor. However, it should also be taken into account that mechanical loads are not the only ones that can affect the durability and strength of the entire structure. Environmental loads [26,27], such as during thermal or chemical disinfection or the effect of UV radiation, would also have to be taken into consideration. In addition, the tabletop structure is often not only made of composite but also of other materials, as shown for model 4, in which the aluminum plate plays an intermediary role and is bonded to the bottom by default. In this case, there is an adhesive joint [28], which should also be properly designed and tested.

Moving on to the analysis of the results, it is important to note their scatter when testing elastic and strength properties. For example, for unidirectional fibers, the value of the relative standard deviation was 6.5% for Young’s modulus, so it should be expected that the comparison of simulation results with the laboratory test will also be subject to error. In the analyzed case for tabletop 4, this difference was 13.53%, which can be considered a satisfactory result because in such a complex construction, taking into account all the details of the structure could cause a significant increase in modeling time and numerical calculations. However, a thoughtful analysis can be made of which factors may affect the differences in results for tabletop 4. The tabletop is mounted to the column by a set of four screws. The column can deform when the bolts are not perfectly rigid, as in the numerical model. In the numerical model, the foam infill and the connection to the aluminum plate were made using “tie” constraints; in reality, there are adhesive joints, which can also deform additionally. There may be structural defects that, for example, result in the tabletop not deforming linearly [29]. It has been observed that as the load increases, the respective differences in displacement increments become larger. Therefore, to be able to make more accurate comparisons and detect structural defects, measurements using digital image correlation DIC [30] for the entire structure are necessary. In general, the deflection results for the demonstrator are greater than in the numerical simulation because the virtual model has no defects and is geometrically perfect.

Ergonomic tabletops will be the next stage in the development of this type of design as they allow the patient’s body to be aligned with the surface, distribute pressure more evenly, and increase comfort. The surgical procedure itself, while in the hospital and on the medical table, is often a stressful situation, hence the aim should be to improve the ergonomics of equipment to make the patient feel comfortable. 

**Figure 19:**
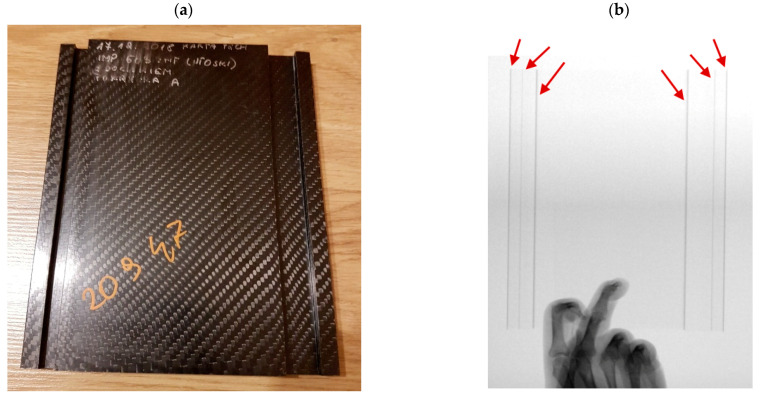
Fragment of the demonstrator with fabricated rail: (**a**) View of a composite part, (**b**) X-ray view (red arrows—vertical walls).

We should also work toward the best possible translucency of the design in terms of X-rays. On the one hand, the use of foam fillings or the use of additional ribs increases stiffness, but on the other, the translucency deteriorates or line disturbances develop where the vertical walls of the laminate are present, as shown in Figure 19 (red arrows).

Such walls can occur, for example, close to the edge, where the rail for attaching accessories is located. However, the topics of accessory mounting and transparency testing will be the subject of future articles.

## 7. Conclusions

In this work, a complete approach to designing, and performing numerical simulations and laboratory tests for medical operating tables was presented. This is the first work of its kind in the world literature on medical tabletops. The following conclusions are drawn from the research.

- The compressive strength of laminates both made of unidirectional fibers and fabrics is more than twice the tensile strength. Compressive strength should be taken into account in research and numerical testing because the tabletop works like a cantilever beam, and thus the lower part of it is compressed.
- The occurring scatter of results in elastic and strength properties leads to a situation in which the result of the numerical simulation will also be subject to error compared to the laboratory test. It is therefore necessary to maintain an appropriate safety factor when designing the structure.
- Three types of ergonomic tops were presented, whose change in cross-section significantly affected the deflection, but the effort of the material remained at a similar level.
- A satisfactory difference (13.53%) was obtained in the results for tabletop 4 between the deflection obtained for the demonstrator and in the numerical simulation.

## Figures and Tables

**Table 1:** Summary of samples used in laboratory tests.

	UD		TW	
	Tension	Compression	Tension	Compression
0°	3 pcs.	3 pcs.	6 pcs.	3 pcs.
45°	3 pcs.	-	6 pcs.	-
90°	3 pcs.	3 pcs.	-	-

**Table 2:** Results from the laboratory and numerical tests.

Deflection	Load [kg]									
	5	10	15	20	25	30	35	40	45	50
Lab. test [mm]	3.71	7.67	11.17	15.88	19.06	24.37	28.54	33.22	37.90	42.56
Abaqus [mm]	3.68	7.35	11.05	14.71	18.5	22.07	25.77	29.44	33.11	36.8
Difference [%]	0.81	4.17	1.07	7.37	2.94	9.44	9.71	11.38	12.64	13.53

## Data Availability

The data presented in this study are available on reasonable request from the corresponding author.

## Appendix B

Summary of the results obtained for the materials used in the study:

(1) KORDCARBON-CPREG-200-UD-3K-EP-120-38 (UD),

(2) KORDCARBON-CPREG-245-T-3K-EP1.5-42-T (TW).

**Property**	**Symbol**	**Unit**	**Value**	
			**(1) UD**	**(2) TW**
Young’s modulus in the direction of fiber orientation (warp direction for TW)	E_t0_	GPa	121.965	48.069
Young’s modulus transverse to the direction of fiber orientation	E_t90_	GPa	7.817	-
Kirchhoff’s modulus	G_12_	GPa	3.434	2.652
Poisson’s ratio	ν_12_	-	0.298	0.064
Tensile strength in the fiber direction (warp direction for TW)	X_t_	MPa	2003.119	453.385
Tensile strength in the direction perpendicular to the fibers	Y_t_	MPa	45.12	-
Shear strength	S	MPa	58.749	58.73
Young’s modulus in the direction of fiber orientation (warp direction for TW)	E_c0_	GPa	111.564	43.4
Young’s modulus transverse to the direction of fiber orientation	E_c90_	GPa	8.094	-
Compressive strength in the fiber direction (warp direction for TW)	X_c_	MPa	793.504	208.99
Compressive strength in the direction perpendicular to the fibers	Y_c_	MPa	131.592	-
